# High-throughput gadobutrol-enhanced CMR: a time and dose optimization study

**DOI:** 10.1186/s12968-017-0400-4

**Published:** 2017-11-06

**Authors:** Tommaso D’Angelo, Chrysanthos Grigoratos, Silvio Mazziotti, Konstantinos Bratis, Faraz Pathan, Alfredo Blandino, Elen Elen, Valentina O. Puntmann, Eike Nagel

**Affiliations:** 1 Department of Biomedical Sciences and Morphological and Functional Imaging, G. Martino University Hospital Messina, Via Consolare Valeria, 1, 98100 Messina, Italy; 2 Institute for Experimental and Translational Cardiovascular Imaging, DZHK Centre for Cardiovascular Imaging, University Hospital Frankfurt, Theodor-Stern- Kai 7, Frankfurt am Main, Germany; 3 G. Monasterio CNR-Tuscany Foundation, Pisa, Italy; 4 Department of Cardiovascular Imaging, King’s College London, Lambeth Wing, St. Thomas’ Hospital, London, UK; 5 Department of Cardiology, Menzies Institute for Medical Research, University of Tasmania, Hobart, Australia; 6 Department of Cardiology, National Cardiovascular Center Harapan Kita, Universitas Indonesia, Jakarta, Indonesia; 7 Department of Cardiology, University Hospital Frankfurt, DZHK Rhein-Main, Theodor-Stern- Kai 7, Frankfurt am Main, Germany

**Keywords:** Cardiovascular magnetic resonance, Gadobutrol, Dose optimization, Time optimization

## Abstract

**Background:**

Reducing time and contrast agent doses are important goals to provide cost-efficient cardiovascular magnetic resonance (CMR) imaging. Limited information is available regarding the feasibility of evaluating left ventricular (LV) function after gadobutrol injection as well as defining the lowest dose for high quality scar imaging. We sought to evaluate both aspects separately and systematically to provide an optimized protocol for contrast-enhanced CMR (CE-CMR) using gadobutrol.

**Methods:**

This is a prospective, randomized, single-blind cross-over study performed in two different populations. The first population consisted of 30 patients with general indications for a rest CE-CMR who underwent cine-imaging before and immediately after intravenous administration of 0.1 mmol/kg body-weight of gadobutrol. Quantitative assessment of LV volumes and function was performed by the same reader in a randomized and blinded fashion. The second population was composed of 30 patients with indication to late gadolinium enhancement (LGE) imaging, which was performed twice at different gadobutrol doses (0.1 mmol/kg vs. 0.2 mmol/kg) and at different time delays (5 and 10 min vs. 5, 10, 15 and 20 min), within a maximal interval of 21 days. LGE images were analysed qualitatively (contrast-to-noise ratio) and quantitatively (LGE%-of-mass).

**Results:**

Excellent correlation between pre- and post-contrast cine-imaging was found, with no difference of LV stroke volume and ejection fraction (*p = 0.538* and *p = 0.095*, respectively). End-diastolic-volume and end-systolic-volume were measured significantly larger after contrast injection (*p = 0.008* and *p = 0.001*, respectively), with a mean difference of 3.7 ml and 2.9 ml, respectively. LGE imaging resulted in optimal contrast-to-noise ratios 10 min post-injection for a gadobutrol dose of 0.1 mmol/kg body-weight and 20 min for a dose of 0.2 mmol/kg body-weight. At these time points LGE quantification did not significantly differ (0.1 mmol/kg: 11% (16.4); 0.2 mmol/kg: 12% (14.5); *p = 0.059*), showing excellent correlation (*ICC = 0.957*; *p < 0.001*).

**Conclusion:**

A standardized CE-CMR rest protocol giving a dose of 0.1 mmol/kg of gadobutrol before cine-imaging and performing LGE 10 min after injection represents a fast low-dose protocol without significant loss of information in comparison to a longer protocol with cine-imaging before contrast injection and a higher dose of gadobutrol. This approach allows to reduce examination time and costs as well as minimize contrast-agent exposure.

**Electronic supplementary material:**

The online version of this article (10.1186/s12968-017-0400-4) contains supplementary material, which is available to authorized users.

## Background

Contrast-enhanced cardiovascular magnetic resonance (CE-CMR) is a cornerstone of clinical cardiology [1, 2]. Data acquisition and post-processing are standardized and recommendations on the dose and timing of contrast agent have been provided [3]. These recommendations suggest a dose range of 0.1–0.2 mmol/kg of a gadolinium-based contrast agent (GBCA) for scar imaging using late gadolinium enhancement (LGE), and recommend a waiting time of at least 10 min after injection for acquiring optimal LGE images. Moreover, despite not specifically stated, the same recommendations show that left ventricular (LV) function should be assessed prior to contrast injection [3]. However, to optimize costs and resource utilization as well as to minimize risks for the patient, there is a drift towards the use of macrocyclic GBCAs at their lowest possible dose [4–8]. Bruder et al. recently reported that a mean dose of 0.12 mmol/kg was used in an analysis based on 37,788 patients undergoing CE-CMR with different types of GBCAs [9]. In addition, an increasing number of centres tend to acquire LV function images after contrast agent injection prior to LGE imaging, thus reducing the total scan time of their imaging protocol, as reflected in a large number of scientific manuscripts [10]. Finally, these trending approaches in routine CMR create two major issues with only limited information available in the literature:

1. Are LV volumes and the subsequently derived LV functional parameters similar before and after contrast injection?
2. Are low doses of macrocyclic contrast agent sufficient to visualize and quantify ischemic and non-ischemic myocardial scar and what is the best time-point for LGE imaging after contrast injection?

The goal of the current study is to address these points systematically and specifically for gadobutrol CE-CMR.

## Methods

All studies were conducted in compliance with the requirements for good clinical practice after approval from the local ethics committee. Each participant provided written informed consent.

### Part 1: Assessment of LV volumes and function before and after contrast agent injection

#### Patient population

Thirty consecutive patients [19 men;43 ± 13 years (27–75 years)] who underwent routine CE-CMR for a rest study were included. Indications were viability assessment after myocardial infarction (*n* = 10), scar detection in chronic coronary disease (*n* = 4), cardiomyopathy (*n* = 9) and suspected myocarditis (*n* = 7).

#### CMR protocol

All CMR examinations were performed on a 1.5 T scanner (Achieva, Philips Healthcare, Best, The Netherlands). A standardized balanced steady state free precession (bSSFP) cine-sequence was performed in combination with parallel imaging (Sensitivity Encoding, factor 2) and retrospective cardiac gating during expiratory breath-hold (TE/TR/flip-angle: 1.7 ms/3.4 ms/60°; spatial resolution: 1.8 × 1.8x8mm). A stack of images covering the whole heart was obtained along the short-axis plane, before and immediately after intravenous administration of 0.1 mmol/kg body-weight of gadobutrol (Gadovist®, Bayer, Leverkusen, Germany), injected at a rate of 3 ml/s, followed by a 20 ml saline flush, using the same slice position.

#### CMR analysis

Cine-bSSFP loops were randomized (crossover design), presented anonymously and displayed for review. Quantitative assessment of all data was performed by the same blinded reader, with 8 years of experience in cardiovascular imaging. Analysis was performed using QMass semi-automated software (v8.1, Medis BV, Leiden, The Netherlands). Endocardial and epicardial contours were drawn in end-systolic and end-diastolic phases for the assessment of functional parameters, which were normalized to the body-surface-area (BSA) [end-systolic volume (ESV), end-diastolic volume (EDV)]. Stroke volume (SV) and ejection fraction (EF) were calculated using standard formulae.

#### Statistical analysis

All statistical analyses were performed using dedicated software (SPSS Statistics, v23, International Business Machines, Inc. Armonk, New York, USA). Shapiro-Wilk test and histograms were preliminarily performed to assess distribution of quantitative data, which presented normal distribution and were expressed as mean ± SD. The significance of differences (*p*) was analysed with paired samples *t-*tests. A value of *p < 0.05* was considered significant. The differences in measurements of LV volumes before and after contrast administration were evaluated by means of Bland-Altman plots and correlation was assessed by intraclass correlation coefficient (*ICC*) statistics. *ICC* results were interpreted as follows: *ICC* < 0.40 = poor correlation, *ICC* 0.41–0.59 = fair correlation, *ICC* 0.60–0.74 = good correlation, *ICC* > 0.75 = excellent correlation [11].

### Part 2: Comparison of gadobutrol 0.1 mmol/kg and 0.2 mmol/kg for detection and quantification of myocardial LGE

#### Patient population

Thirty patients [23 men;60 ± 14 years (range: 27–80)] who underwent routine CMR for a contrast-enhanced rest study were included. Fifteen patients with known myocardial infarction [11 men;69 ± 9 years (range: 51–80)] and 15 patients with known diagnosis of hypertrophic cardiomyopathy (HCM) [12 men;51 ± 12 years (range: 27–68)] were prospectively enrolled to obtain patients with ischemic and patients with non-ischemic scar. Exclusion criteria were the administration of any contrast agent in the prior 72 h, a history of any severe allergic reaction or hypersensitivity to contrast media, eGFR <60 ml/min and general contraindications to CMR.

#### CMR protocol

All examinations were performed on a 1.5 T MR scanner scanner (Achieva, Philips Healthcare). Standardized LGE imaging was performed using 2D turbo-field-echo inversion recovery T1-weighted sequence (TE/TR/flip-angle: 2 ms/3.4 ms/25°; spatial resolution: 1.4 × 1.4x8mm) with an individually adapted prepulse delay to achieve optimal nulling of the normal myocardium. For each patient, the first LGE imaging study was performed by acquiring a stack of images covering the whole heart along the short-axis plane at 5, 10, 15, and 20 min after peripheral bolus injection of 0.2 mmol/kg body-weight (*double-dose*) of gadobutrol (Gadovist®, Bayer), injected at a rate of 3 ml/s and followed by a 30 ml saline chaser. Each subject repeated LGE imaging after a minimum of 6 days (range: 6–21, median 8), by acquiring the same stack of images along the short-axis plane after peripheral bolus injection of 0.1 mmol/kg body-weight (*single-dose*) of gadobutrol, with same injection rate (3 ml/s) and followed by a saline flush (20 ml at 3 ml/s). To minimize the burden on the patients and to reduce the research scan time of the second examination, images were acquired only twice (i.e. 5 and 10 min after contrast injection).

#### CMR analysis

LGE images were randomized (crossover design), presented anonymously and evaluated in consensus by a radiologist and a cardiologist, respectively with 5 and 15 years of experience in cardiovascular imaging. All analyses were done using QMass (v8.1, Medis BV). LGE quantification was performed by the full width at half maximum (FWHM) method, which uses half of the maximal signal intensity (*SI*) within the scar as the threshold, and reported as a percentage of total LV mass [12]. In addition, for each patient, ROIs were drawn by the readers in the normal myocardium (*MYO*), in LGE areas, in LV cavity (*LVC*) and in air (*A*) outside the patient’s body, in order to evaluate the signal-to-noise ratio (*SNR*) and contrast-to-noise ratio (*CNR*). SNR was calculated for remote myocardium (*SNR*_*MYO*_), LGE areas (*SNR*_*LGE*_) and LV cavity (*SNR*_*LVC*_), by dividing each respective mean SI for standard deviation of air (*SD*_*A*_). CNR values between LGE areas and normal myocardium (*CNR*_*LGE-MYO*_) and between LGE areas and LVC (*CNR*_*LGE-LVC*_) were obtained in order to assess optimal scar delineation within the myocardium and subendocardial area respectively. The following formulas were used:

$$ {CNR}_{LGE- MYO}=\left({SI}_{LGE}\hbox{--} {SI}_{MYO}\right)/{SD}_A\kern0.5em and\kern0.5em {CNR}_{LGE- LVC}=\left({SI}_{LGE}\hbox{--} {SI}_{LVC}\right)/{SD}_A $$

#### Statistical analysis

All statistical analyses were performed using dedicated software (SPSS Statistics, v23; International Business Machines, Inc.). Shapiro-Wilk test and histograms were performed to test for normal distribution of the data. Normal distributed data was expressed as mean and standard deviation, non-normal distribution as medians and interquartile ranges (*IQR*). Comparison between groups was performed with Wilcoxon signed-rank test for paired data. Comparison across multiple groups was assessed using non-parametric analysis of variance (i.e. Kruskal-Wallis test). In particular, Wilcoxon signed-rank test was used to obtain *p*-*values* for 0.1 mmol/Kg protocol. Kruskal-Wallis test was used to obtain *p*-*values* for 0.2 mmol/Kg protocol, due to the presence of four different time-points; an additional Wilcoxon signed-rank test was performed to assess any significant differences between 15 and 20 min time delays. A value of *p < 0.05* was considered statistically significant. The differences in measurements were evaluated by means of Bland-Altman plots and correlation was assessed by intraclass correlation coefficient (*ICC*) statistics. *ICC* results were interpreted as follows: *ICC* < 0.40 = poor correlation, *ICC* 0.41–0.59 = fair correlation, *ICC* 0.60–0.74 = good correlation, *ICC* > 0.75 = excellent correlation [11].

## Results

### Part 1: Assessment of LV volumes and function before and after contrast agent injection

**Fig. 1:**
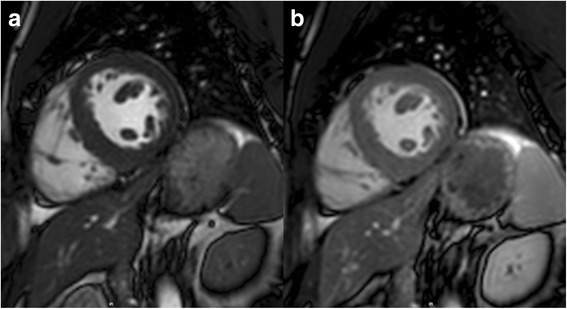
Balanced steady state free precession short-axis images obtained at the mid ventricular level before (**a**) and after (**b**) injection of gadobutrol 0.1 mmol/kg body-weight

EDV and ESV were measured significantly larger after contrast injection in comparison to native images (*p = 0.008* and *p = 0.001*, respectively). This difference was not significant for the derived parameters SV and EF (*p = 0.538* and *p = 0.095*, respectively). There was excellent correlation between pre- and post-contrast LV volumes and EF (all *ICC ≥ 0.895*; *p < 0.001*). Bland-Altman analysis showed a mean difference for post-contrast measurements of 3.7 ml and 2.9 ml, respectively for EDV and ESV (Fig. 1 and Table 1).

**Table 1:** Left ventricular (LV) structure and functional values obtained before (*C-*) and after (*C+*) injection of gadobutrol 0.1 mmol/kg body-weight using a cine-balanced steady state free precession (bSSFP) sequence and intra-observer agreement

	C+	C-		*p*-value	ICC*	Mean difference (LOA)
EDV *(ml)*	142.1 ± 39.8	138.4 ± 39.7	C+ vs. C-	*0.008*	0.980	3.7 ml (17.9, −10.4)
ESV *(ml)*	67.9 ± 29.5	65 ± 28.8		*0.001*	0.986	2.9 ml (10.8, −5.1)
SV *(ml)*	74.2 ± 17.1	73.4 ± 15.5		0.538	0.895	0.9 ml (15.6, −13.9)
EF *(%)*	53.8 ± 9.4	54.8 ± 8.8		0.095	0.935	−1% (5.3, −7.3)

Values expressed as mean ± SD; significant values are in bold; *p < 0.001 for all ICC values

*EDV*, End-diastolic volume, *EF* Ejection fraction, *ESV* End-systolic volume, *LOA* Limits of agreement, *SV* Stroke volume

### Part 2: Comparison of gadobutrol 0.1 mmol/kg and 0.2 mmol/kg for detection and quantification of myocardial LGE

**Fig. 2:**
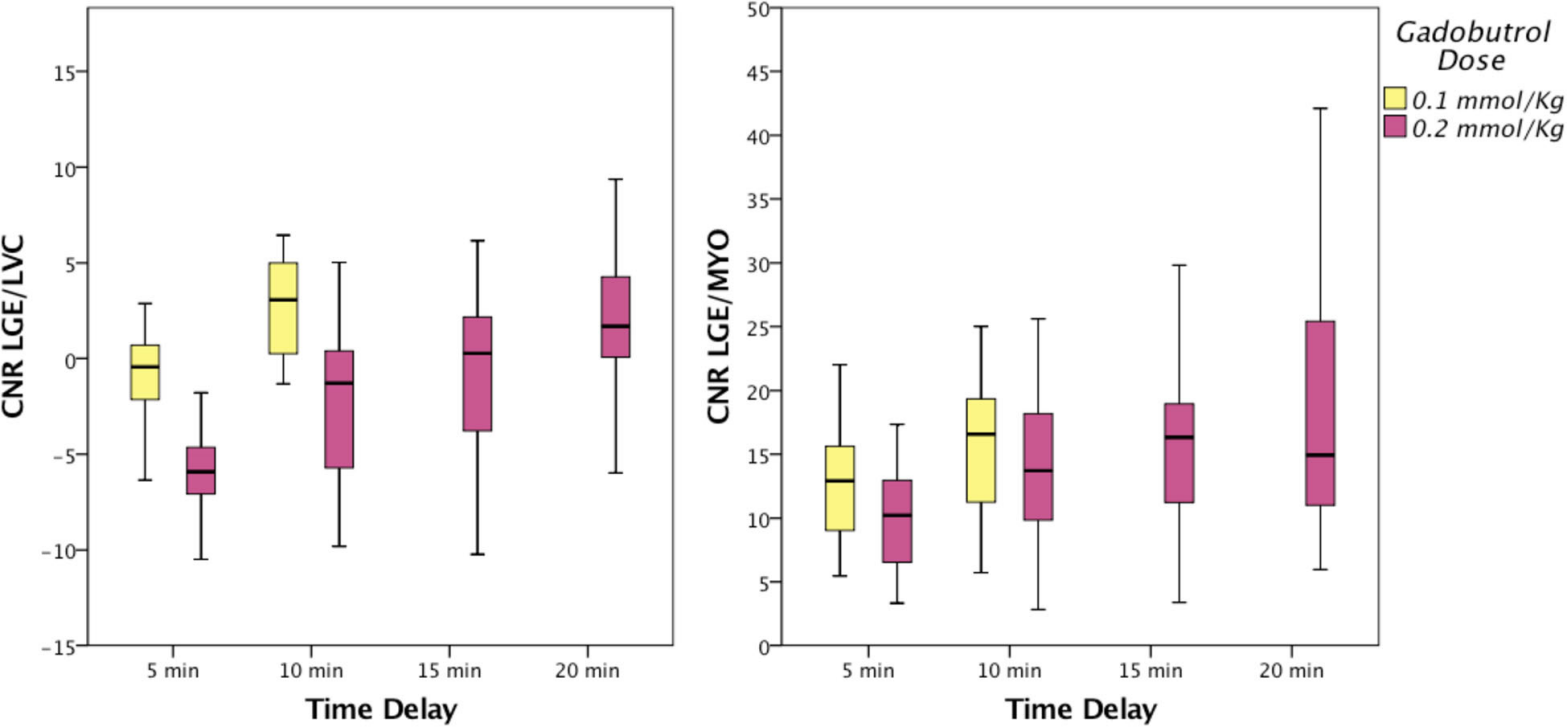
Graphs showing contrast-to-noise-ratio (*CNR*) between late gadolinium enhancement (*LGE)* scar and left ventricular cavity (*LVC*) and between *LGE* scar and remote myocardium (*MYO*) measured at different time delays for 0.1 mmol/kg (yellow) and for 0.2 mmol/kg body-weight protocol (purple)

**Fig. 3:**
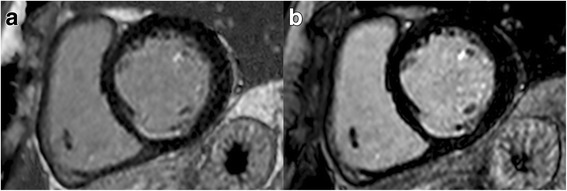
2D turbo-field-echo inversion recovery T1-weighted basal short-axis images showing a subendocardial scar in inferoseptal segment. Good contrast between subendocardial border and LV blood pool is obtained after intravenous injection of gadobutrol 0.1 mmol/kg with a time delay of 10 min (**a**) as well as after injection of a 0.2 mmol/kg dose with a time delay of 20 min (**b**)

*CNR*_*LGE-MYO*_ and *CNR*_*LGE-LVC*_ were optimal at 10 min for *single-dose* protocol, and at 20 min for *double-dose* protocol (Figs. 2 and 3). At these time-points a trend towards higher values of absolute CNR with the *single-dose* protocol was found in the Bland-Altman plots but without significant differences of *CNR*_*LGE-MYO*_ and *CNR*_*LGE-LVC*_ (*p = 0.668* and *p = 0.525*) (Table 2). At all earlier time-points (5, 10 and 15 min post-injection) *CNR*_*LGE-LVC*_ of 0.2 mmol/kg protocol was significantly inferior (*p < 0.001* at 5 and 10 min, and *p = 0.002* at 15 min).

**Table 2:** Comparison of CNR values for LGE imaging obtained at the best time delays for different doses

	0.1 mmol/kg (at 10 min) vs. 0.2 mmol/kg gadobutrol (at 20 min)		0.1 mmol/kg (at 10 min) vs. 0.2 mmol/kg gadobutrol (at 15 min)	
	*p*-value	Mean difference (LOA)	*p*-value	Mean difference (LOA)
SNR _LVC_	0.075		**<0.001**	
SNR _MYO_	0.405		0.600	
SNR _LGE_	0.371		0.861	
CNR _LGE-LVC_	0.750	3 (8.1, −2.1)	**0.002**	4.3 (9.7, −1.1)
CNR _LGE-MYO_	0.237	4.7 (14.3, −4.9)	0.877	4 (13.7, −5.7)

Significant values are in bold, *CNR* Contrast-to-noise ratio, *LGE* Late gadolinium enhancement, *LOA* Limits of agreement, *LVC* Left ventricular cavity, *MYO* Myocardium, *SNR* Signal-to-noise ratio

The extent of LGE, expressed in terms of percentage of involvement of myocardial mass, did not significantly differ between the two protocols at their optimal time points (*single-dose*: 11% (16.4); *double-dose*: 12% (14.5); *p = 0.059*), showing excellent correlation (*ICC = 0.957; p < 0.001*).

The diagnostic performance of LGE quantification in ischemic and non-ischemic conditions did not significantly differ between the two doses (Table 3). In particular, in the ischemic group, percentage of LGE was 19% (17) and 19.8% (20.2) respectively for the *single-* and *double-dose* protocol (*p = 0.248*), and agreement was excellent (*ICC = 0.967; p < 0.001*). In the non-ischemic group, values were respectively of 7% (6.7) and 10.3% (8.4) (*p = 0.244*; *ICC = 0.876; p < 0.001*). Extent of LGE similarly did not differ between *single-dose* at 10 min and *double-dose* at 15 min (see Additional file 1).

**Table 3:** Late gadolinium enhancement quantitative results at best time-delays for different gadobutrol doses and its relative comparison

*Gadobutrol Dose*	*0.1 mmol/kg*	*0.2 mmol/kg*		*0.2 mmol/kg (at 20 min)* vs. *0.1 mmol/kg (at 10 min)*		
*Time delay*	*10 min*	*20 min*	*COMPARISON*	*p-value*	ICC*	*mean difference (LOA)*
LGE mass *(all patients)*	11% (16.4)	12% (14.5)		0.059	0.957	0.9% (7, −5.2)
LGE mass *(CAD patients only)*	19% (17)	19.8% (20.2)		0.248	0.967	0.2% (6.3, −5.8)
LGE mass *(HCM patients only)*	7% (6.7)	10.3% (8.4)		0.244	0.876	1.6% (7.6, −4.4)

* *p* < 0.001 for all ICC values, *CAD* Coronary artery disease, *HCM* Hypertrophic cardiomyopathy, *LGE* Late gadolinium enhancement, *LOA* Limits of agreement

## Discussion

**Fig. 4:**
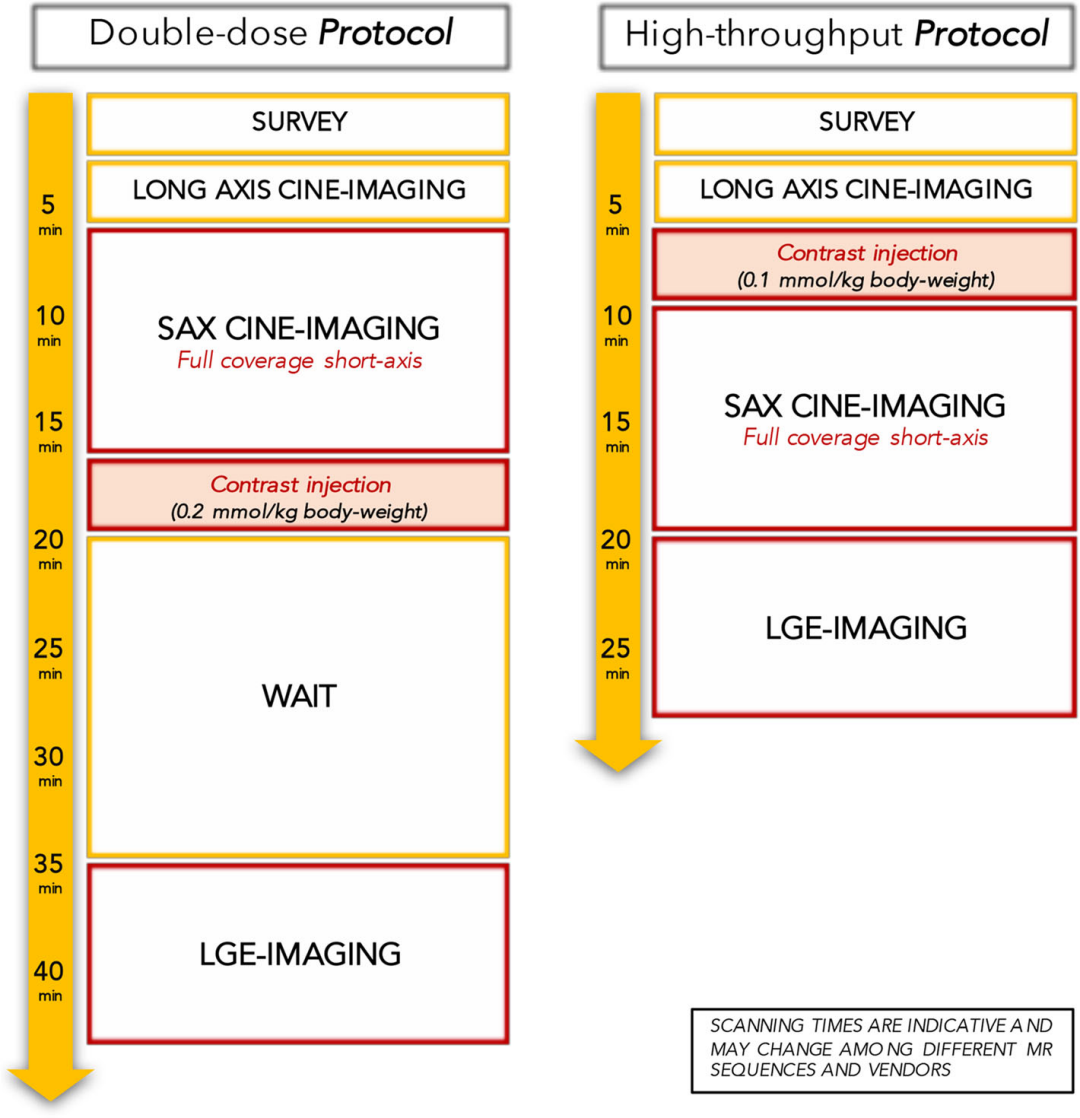
Illustration showing the advantages in terms of time-saving of our recommended high-throughput CE-CMR protocol (gadobutrol 0.1 mmol/kg body-weight) over a conventional CE-CMR protocol performed using a gadobutrol dose of 0.2 mmol/kg body-weight

Our data show two important results that may allow routine use of low doses of GBCA in a high-throughput protocol. First, the assessment of LV function can be performed *after* contrast agent injection as SV and EF are not influenced by contrast agent administration. A small – not clinically relevant – bias can be observed for the quantification of EDV and ESV, which needs to be taken into consideration for scientific follow-up studies. We also demonstrate that scar imaging with LGE can be performed with a dose of 0.1 mmol/kg of gadobutrol, having similar SNR, CNR and extent of scar as using a dose of 0.2 mmol/kg. Importantly, contrast between blood pool and scar at an earlier time-point (i.e. a time delay of 10 min) is better with the *single-dose* rather than *double-dose*, which requires an interval of at least 20 min to wash out the blood pool signal. The combination of these two findings allows a significant shortening of a standardized CE-CMR protocol, which may eventually include native tissue characterization (e.g. T2- and T2*-weighted imaging, T1-mapping) followed by gadobutrol injection at a dose of 0.1 mmol/kg, assessment of LV structure/function and, 10 min after contrast-injection, LGE imaging (Fig. 4) [3].

A few points require discussion. bSSFP-cine sequence is the most widely used for assessing LV function and structure, since its signal depends on the T2/T1 ratio and is relatively independent of inflow effects. This technique provides optimal CNR, allowing for accurate contour detection of the epicardial and endocardial borders [13]. In our study, LV EF and SV were identical before and after contrast administration, while a small systematic difference was found for LV EDV and ESV (3.7 ml and 2.9 ml respectively). While similar observations have been reported for LV EF and SV [14] previous studies have not reported on EDV and ESV independently, demonstrating a small but significant bias for EDV and ESV between pre- and post-contrast cine-bSSFP. This difference is clinically rather irrelevant and within the usual inter-observer and inter-study variability [15]. However, it may be of importance for scientific follow-up studies which include some examinations with contrast agent and some without.

In CE-CMR contrast dose and delivery need to be accurately chosen for reliable quantitative analysis since SNR of scar tissue versus normal myocardium varies depending on the type of GBCA as well as the dose and time after injection [16, 17]. Gadobutrol has been proven to allow for adequate LGE imaging at doses of 0.2 mmol/kg [18, 19], 0.15 mmol/kg [20, 21], and 0.1 mmol/kg [22, 23]. In each of these studies, gadobutrol was compared to different GBCAs, but not as a dose-finding study using the same contrast agent.

A common problem of LGE imaging is the difficulty to differentiate subendocardial scar from LV blood-pool [24]. A possible solution is based on black blood scar imaging [25] or a longer wait-time between contrast injection and data acquisition [26]. Our data show that a lower dose of gadobutrol of 0.1 mmol/kg is sufficient for adequate LGE assessment. In addition, the contrast between blood pool and subendocardial scar using a lower dose of gadobutrol is superior to a higher dose 10 min post-injection. After injection of gadobutrol at a dose of 0.2 mmol/kg a similar CNR between blood and subendocardial scar can only be achieved after 20 min. Given the need for rapid examinations for patient’s benefit as well as for economic reasons, an earlier time-point for LGE is favourable. Importantly, this data holds for ischemic and non-ischemic scar, both for SNR and CNR, as well as for scar burden using an identical threshold. Similar results, in terms of CNR and scar burden, have been obtained by other studies, which observed how a *single-dose* of gadobutrol might be equivalent to *double-dose* of different linear GBCAs [22, 23]. However, while these studies assumed that the feasibility of a *single-dose* might have been related to higher T1-relaxivity of gadobutrol, we are the first to investigate how different doses of the same contrast-agent affect the quality and quantification of LGE imaging.

The results of our two substudies show that time efficient standard imaging can be achieved by performing cine-imaging after injection of 0.1 mmol/kg of gadobutrol and early LGE imaging after 10 min. This rapid protocol is feasible without clinically relevant changes of LV volumes, no change of SV and EF.

We did not test other GBCAs, however, the basic concept (i.e. post-contrast cine-imaging, earlier scar imaging with lower GBCAs dose) should be transferable considering aspects such as albumin binding, different relaxivities and other potential differences. Our data is also limited to rest examinations. While we expect similar results when the contrast agent is given during adenosine stress, we did not explicitly test for this. Stress-perfusion imaging can be well performed with gadobutrol doses of approximately 0.1 mmol/kg and as such stress-perfusion, cine-imaging and LGE imaging might well fit into a short standardized exam [3]. In cases needed, rest perfusion could then be added at the end of the exam. There are certain limitations of our study which need to be addressed. Firstly, our data was obtained with 1.5 T scanner and the results may not apply to 3 T platforms. Secondly, we have not assessed CNR values for time delays longer than 10 min for the 0.1 mmol/kg protocol and longer than 20 min for 0.2 mmol/kg protocol to keep the exam time short. Despite this may hypothetically hide any better CNR values, we believe that reducing the scan time is a core aim to minimize the burden of CMR scanning on the patients and health care systems.

## Conclusion

Based on our data, we provide the scientific evidence to recommend a standardized high-throughput CMR rest protocol giving gadobutrol at a dose of 0.1 mmol/kg before cine-imaging, and performing LGE imaging 10 min after injection. Such a rapid low-contrast dose approach is favourable for patient’s comfort (shorter examination time), patient’s safety (lower dose of GBCA) and economic reasons (lower costs for scan time and contrast media).

## Additional file

Additional file 1:Table S1. Late enhancement qualitative results at the different time delays for different doses of gadobutrol. **Table S2.** Late enhancement quantitative results (percentage of LGE myocardial mass) obtained at 10 min with 0.1 mmol/kg protocol and at 15 min with 0.2 mmol/kg protocol. (DOCX 58 kb)

## Abbreviations

A: Air
BSA: Body-surface-area
bSSFP: Balanced steady state free precession
CE-CMR: Contrast-enhanced cardiovascular magnetic resonance
CNR: Contrast-to-noise ratio
EDV: End-diastolic volume
EF: Ejection fraction
eGFR: estimated glomerular filtration rate
ESV: End-systolic volume
FWHM: Full width half maximum
GBCA: Gadolinium-based contrast agent
HCM: Hypertrophic cardiomyopathy
ICC: Intraclass correlation coefficient
IQR: Interquartile range
LGE: Late gadolinium enhancement
LV: Left ventricle/left ventricular
LVC: Left ventricular cavity
MYO: Myocardium/myocardial
ROI: Region of interest
SD: Standard deviation
SI: Signal intensity
SNR: Signal-to-noise ratio
SV: Stroke volume
