# Supplementary material for: High-throughput gadobutrol-enhanced CMR: a time and dose optimization study
Source: J Cardiovasc Magn Reson. 2017 Nov 6;19:83. doi: 10.1186/s12968-017-0400-4 (PMC5674223; doi:10.1186/s12968-017-0400-4)
Supplement: Additional file 1:Table S1. — Late enhancement qualitative results at the different time delays for different doses of gadobutrol. Table S2. Late enhancement quantitative results (percentage of LGE myocardial mass) obtained at 10 min with 0.1 mmol/kg protocol and at 15 min with 0.2 mmol/kg protocol. (DOCX 58 kb) [file 12968_2017_400_MOESM1_ESM.docx]

**Table S1**

Late enhancement qualitative results at different time delays for different doses of gadobutrol.

| *Gadobutrol Dose* | *0.1 mmol/Kg* | | *0.2 mmol/Kg* | | | |
| --- | --- | --- | --- | --- | --- | --- |
| *Time point* | *5 min* | *10 min* | *5 min* | *10 min* | *15 min* | *20 min* |
| SNR _LVC_ | 14.86 (9) | 14.7 (9.1) | 16.43 (8.77) | 18.87 (11) | 17.97 (11.3) | 16.46 (11.5) |
| SNR _MYO_ | 1.36 (0.9) | 1.44 (1) | 1.58 (0.8) | 1.56 (1) | 1.5 (0.9) | 1.46 (0.9) |
| SNR _LGE_ | 14.14 (6.7) | **17.72 (9.1)** | 11.57 (7.2) | 15.3 (9.8) | **18.35 (9.8)** | 16.22 (15) |
| *CNR _LGE-LVC_* | -0.42 (2.9) | **3.06 (4.8)** | -5.91 (2.5) | -1.27 (6.1) | 0.26 (6.3) | **1.7 (4.5)*** |
| *CNR _LGE-MYO_* | 12.91 (6.8) | **16.57 (8.5)** | 10.39 (6.8) | 13.7 (9.5) | **16.33 (9.5)** | **14.93 (15.3)** |

*values expressed as median (IQR); significant values are in bold; *best CNR_LGE-LVC_ value for 0.2 mmol/kg dose*

**Table S2**

Late enhancement quantitative results (percentage of LGE myocardial mass) at different time delays for different gadobutrol doses and its relative comparison.

| *Gadobutrol Dose* | *0.1mmol/kg* | *0.2 mmol/kg* |  | *0.2 mmol/kg* *(at 15 minutes)*  vs.  *0.1mmol/kg (at 10 minutes)* | | |
| --- | --- | --- | --- | --- | --- | --- |
| *Time point* | *10 min* | *15 min* | *COMPARISON* | *p-value* | *ICC** | *mean difference (LOA)* |
| *LGE mass* *(all patients)* | **11% (16.4)** | 11.9% (14.9) |  | 0.559 | 0.951 | 0.45 % (7.3, -6.4) |
| *LGE mass* *(CAD patients only)* | **19% (17)** | 18.2% (20.5) |  | 0.346 | 0.959 | -0.7 % (6.3, -7.9) |
| *LGE mass* *(HCM patients only)* | **7% (6.7)** | 10.5% (8) |  | 0.073 | 0.872 | 1.7 % (7.7, -4.4) |

** p<0.001 for all ICC values; LOA: limits of agreement*
